# Oropouche Fever, Cuba, May 2024

**DOI:** 10.3201/eid3010.240900

**Published:** 2024-10

**Authors:** Ana Julia Benitez, Mayling Alvarez, Lissette Perez, Rosario Gravier, Silvia Serrano, Denelsys Milagro Hernandez, Melissa Maria Perez, Gladys Gutierrez-Bugallo, Yanet Martinez, Ariamys Companioni, Carilda Peña, Jose Raul de Armas, Dayana Couto, Iliovanys Betancourt I, Madelaine Rivera Sanchez, Sonia Resik, Vivian Kouri, Maria G. Guzman

**Affiliations:** Pedro Kouri Tropical Medicine Institute, Havana, Cuba (A.J. Benitez, M. Alvarez, L. Perez, R. Gravier, S. Serrano, D.M. Hernandez, M. Perez, G. Gutierrez-Bugallo Y. Martinez, A. Companioni, S. Resik, V. Kouri, M.G. Guzman);; Cuban Ministry of Health, Havana (C. Peña, J.R. de Armas, D. Couto, I. Betancourt, M. Rivera)

**Keywords:** Oropouche virus, orthobunyavirus, arboviruses, viruses, vector-borne infections, Cuba

## Abstract

Phylogenetic analyses showed that the virus responsible for a May 2024 Oropouche fever outbreak in Cuba was closely related to viruses from Brazil in 2023. Pools of *Ceratopogonidae* spp. biting midges and *Culex quinquefasciatus* mosquitoes were positive for Oropouche viral RNA. No cases were severe. Virus extension to new areas may increase case numbers and severity.

On May 9, 2024, the Pan American Health Organization reported 5,193 cases of Oropouche fever (also called Oropouche virus disease) in Bolivia, Brazil, Colombia, and Peru and sent an alert with regard to autochthonous cases in areas of Brazil and Bolivia, where the disease had not been previously reported (*1*). During the past decade, Oropouche fever has been reported mainly in the Amazon region. However, in 2014, the first case outside that region was registered in Haiti, raising concerns about its geographic extension (*2*). The virus has been detected in Central and South America (e.g., Bolivia, Brazil, Colombia, Peru, and Panama); ≈500,000 cases have been diagnosed. Given the clinical picture of Oropouche fever (fever, headache, arthralgia, myalgia), the disease can be confused with dengue, Zika, or other febrile illnesses.

Oropouche virus (OROV) is a member of the family *Peribunyaviridae*, genus *Orthobunyavirus*, and was first identified in Trinidad and Tobago. OROV is maintained in nature through sylvatic and urban cycles. The urban cycle is thought to mainly involve the bite of the *Culicoides paraensis* midge. Another urban vector in tropical regions, where it feeds on both humans and animals, is the *Culex quinquefasciatus* mosquito (*3*). The OROV genome comprises 3 single-stranded negative-sense RNA segments (large, medium, and small). Sequencing studies of the small segment suggest the existence of 4 genotypes (I–IV) (*4*,*5*).

The source of patients for dengue surveillance in Cuba is the primary healthcare level through the search, clinical management, and notification of acute febrile illness (AFI) cases of unknown etiology or cases suspected of being dengue. A network of 30 laboratories with capacity for dengue IgM detection guarantees serologic surveillance conducted on samples collected at day 6 of fever onset. Positive samples are confirmed at the National Reference Laboratory of the Institute “Pedro Kouri” (NRL-IPK). Molecular surveillance at the NRL-IPK enables real-time PCR identification of dengue viruses in acute-phase samples from patients with suspected dengue cases. Negative samples are tested for Zika, chikungunya, and yellow fever viruses. The NRL-IPK is also in charge of virus genetic surveillance (*6*). Environmental and entomologic surveillance complement the national integrated arbovirus surveillance system.

## The Study

On May 20, 2024, the NRL-IPK received samples from 89 patients with AFI of unknown etiology in Santiago de Cuba and Cienfuegos Provinces, Cuba, where an increase of similar cases had been reported, most negative for dengue IgM. Patients reported acute fever onset with headache and joint pain for 2–3 days and rapid recovery. Median patient age was 35 years (interquartile range [IQR] 19–50); 49.4% of patients were female and 51.6% male. Of the 89 serum samples received, 69 were from Santiago de Cuba (Boniato, n = 39; Songo la Maya, n = 30) and 20 from Cienfuegos (Abreu, n = 4; Cienfuegos, n = 11; and Rodas, n = 5). Urine (n = 6) and feces (n = 30) samples were also collected from the 89 patients.

We extracted RNA by using the commercial QIAamp Viral RNA Mini Kit (QIAGEN, https://www.qiagen.com) according to the manufacturer’s instructions. We processed extracted RNA for dengue, Zika and chikungunya viruses using by real-time PCR (*7*–*9*), and all serum samples were negative. Rapid test results for dengue nonstructural protein 1 (Bioline Dengue Duo; Abbott Laboratories, https://www.abbott.com) and chikungunya IgM (Kabla Diagnósticos, https://www.biodiagnosticos.com) were also negative. Fecal samples were negative for enterovirus (*10*).

We examined 89 serum and 6 urine samples for Oropouche and Mayaro viruses by using real-time PCR (*11*). All samples were negative for Mayaro virus, and 74 (83. 1%) serum samples (54 from Santiago de Cuba and 20 from Cienfuegos) and 5 (83.3%) urine samples were positive for OROV.

**Figure 1:**
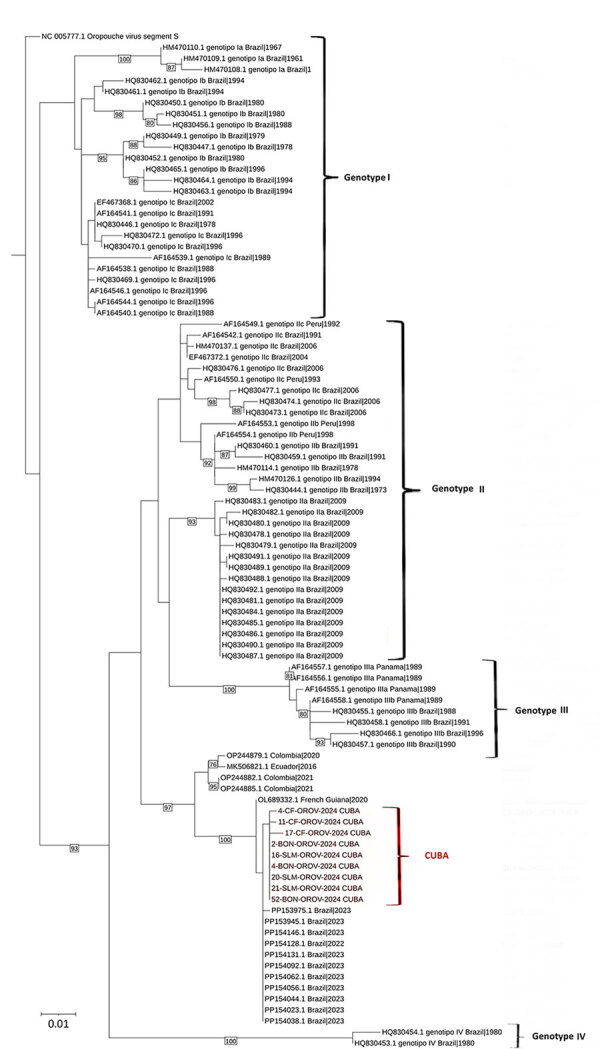
Molecular phylogenetic analysis of Oropouche viruses isolated in Cuba (red bracket) and reference sequences. The evolutionary history was inferred by using the maximum-likelihood method based on the Kimura 2-parameter model to the small segment of Oropouche orthobunyavirus from 9 patients from Boniato, Songo La Maya, and Cienfuegos (PP921382, PP921383, PP921384, PP921385, PP921386, PP921387, PP921388, PP921389, PP921390) (*14*). The tree with the highest log likelihood (−2,403.4997) is shown. The percentage of trees in which the associated taxa clustered together is shown next to the branches. Initial tree(s) for the heuristic search were obtained by applying the neighbor-joining method to a matrix of pairwise distances estimated by using the maximum composite likelihood approach. A discrete gamma distribution was used to model evolutionary rate differences among sites (5 categories [+G, parameter = 0.1407]). The tree is drawn to scale, with branch lengths measured in number of substitutions per site. The analysis involved 101 nt sequences deposited in GenBank (accession numbers shown) from the different outbreaks and genotypes of Oropouche virus in the Americas and the Caribbean region. All positions with <95% site coverage were eliminated (i.e., <5% alignment gaps, missing data, and ambiguous bases were allowed at any position) The final dataset contained 521 positions. Evolutionary analyses were conducted in MEGA6 (*13*).

For the viral genetic characterization, we studied 9 serum samples (3 from Boniato, 3 from Songo La Maya, and 3 from Cienfuegos). We subjected extracted RNA to reverse transcription by PCR using the QIAGEN OneStep RT-PCR Kit system and primers NORO5 (5′-AAAGAGGATCCAATAATGTCAGAGTTCATTT-3′) and ORON3 (5′-AATTCGGAATTGGCATATAGTGGAATTCAC-3′) (*12*). We purified the 626-bp fragments obtained, which encode for the viral nucleocapsid (positions 85–718), by using the QIAquick PCR Purification Kit (QIAGEN), followed by sequencing with the Dye Terminator Cycle Sequencing Quick Start Kit (Analis, https://www.analis.com) and primers NORO5, ORON3, OROV-F (5′-TCCGGAGGCAGCATATGTG-3′), and OROV-R (5′-AAGTGCTCAATGCTGGTGTTGT-3′) (donated by the Pan American Health Organization). We purified the sequencing products and then separated the generated fragments on a CEQ 8800 Genetic Analysis System sequencer (Analis). We edited and assembled the electropherograms by using Sequencher version 4.10.1 software (Gene Codes Corporation, https://www.genecodes.com). As a reference, we used the complete sequence of the small segment of the Oropouche orthobunyavirus strain (GenBank accession no. OP244879.1, Oropouche orthobunyavirus isolate 0200178W, small segment, complete sequence). Phylogenetic analyses (MEGA version 6) (*13*) revealed that the isolated viruses clustered in a separate branch, closely related to those reported from Brazil in 2023 (Figure 1).

After we had identified OROV transmission, national surveillance for dengue and Oropouche fever was intensified (active AFI case search). In addition, vector control intervention (adulticide treatment, source reduction, environmental management, biolarvicide application on permanent breeding sites) was applied in the areas with confirmed transmission.

**Figure 2:**
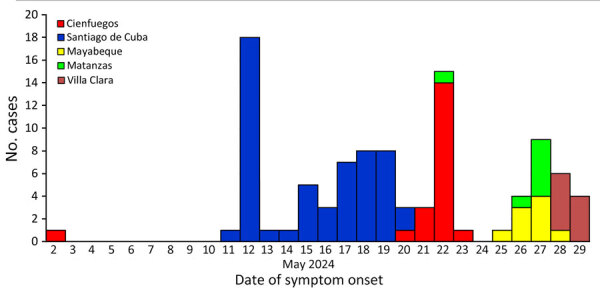
Confirmed Oropouche cases according to date of onset of signs/symptoms and provinces, Cuba, May 2024.

During May 28–June 3, 2024, we processed 31 serum samples collected from locations with increased AFI cases (San Nicolas de Bari in Mayabeque; Perico, Cienaga de Zapata, and Jovellanos in Matanzas; and Ranchuelo in Villa Clara). OROV infection was confirmed in 25 (80.6%) samples: 7 from Matanzas, 9 from Mayabeque, and 9 from Villa Clara Provinces (Table 1). Median patient age was 34.5 years (range 4–83 years), male:female ratio was 1.1. Signs and symptoms by order of frequency were fever, headache, general malaise, myalgia, and arthralgia (Table 2; Figure 2; Appendix Table). No serious or fatal cases were reported.

**Table 1:** Epidemiologic characteristics of confirmed cases of Oropouche fever, Cuba, 2024*

Data	Values
M:F ratio	51/48 (1.1)
Median days of sample collection according to illness onset (range)	2 (0–4)
Median age, y (range) [IQR]	34.5 (4–83) [18–50.5]

*IQR, interquartile range.

**Table 2:** Clinical characteristics of patients with confirmed Oropouche fever, Cuba, 2024

Clinical signs/symptoms	No. (%) patients
Fever	86 (86.9)
Headache	71 (71.7)
General malaise	51 (51.5)
Arthralgia	22 (22.2)
Asthenia	18 (18.1)
Anorexia	16 (16.2)
Retroocular pain	14 (14.1)
Abdominal pain	8 (8)
Vomiting	7 (7)
Diarrhea	7 (7)
Chills	4 (4)
Lumbar pain	3 (3)

To investigate the vector involved with OROV transmission in Cuba, we collected 156 *C. quinquefasciatus* mosquitoes and 49 *Ceratopogonidae* spp. from suspected areas of transmission in 5 blocks of houses in Boniato. After potential vector identification, we grouped not visibly engorged female *C. quinquefasciatus* mosquitoes into 9 pools (10–20/pool) and *Ceratopogonidae* spp. specimens into 2 pools (7–19/pool). We extracted RNA and conducted real-time PCR. Positive samples were attributed to 5 (56%) pools of *C. quinquefasciatus* mosquitoes and 1 (50%) pool of *Ceratopogonidae* biting midges.

Among our study limitations was collection of signs/symptoms by using a standardized form for AFI or dengue cases; consequently, we might have missed less common presentations such as meningitis. In addition, the presence of viral RNA does not confirm the vector role in virus transmissibility. More work is needed to determine the primary vectors responsible for the current outbreak.

## Conclusions

Oropouche fever is an emerging disease of concern in South and Central America. Transmission outside of the Amazon region is probably silent and not detected by public health surveillance systems. Our results confirm an outbreak of Oropouche fever in Cuba. Transmission was detected in semi-urban localities of 5 of 16 provinces located in the western, central, and eastern parts of the country. Dengue surveillance enabled us to identify cases with nondengue AFI. As of August 2024, OROV transmission had been confirmed in 7 provinces. The outbreak in Cuba alerts the Americas and the world of the need for integrated dynamic surveillance systems to detect the introduction and early transmission of OROV and consequently implement effective measures for its control.

Appendix Additional information for Figure 2.
