# Supplementary material for: Oropouche Fever, Cuba, May 2024
Source: Emerg Infect Dis. 2024 Oct;30(10):2155–9. doi: 10.3201/eid3010.240900 (PMC11431908; doi:10.3201/eid3010.240900)
Supplement: Appendix — Additional information for Figure 2. [file 24-0900-Techapp-s1.pdf]

Article DOI: https://doi.org/10.3201/eid3010.240900

EID cannot ensure accessibility for supplementary materials supplied by authors. Readers who have difficulty accessing supplementary content should contact the authors for assistance.

Oropouche Fever, Cuba, May 2024

Appendix

Appendix Table. Confirmed Oropouche cases according to date of onset of symptoms and provinces. Cuba, May 2024

Day of illness onset,	Provinces					
day-mo-yr	Cienfuegos	Matanzas	Mayabeque	Santiago de Cuba	Villa Clara	Total confirmed*
02-05-2024	1					1
03-05-2024						
04-05-2024						
05-05-2024						
06-05-2024						
07-05-2024						
08-05-2024						
09-05-2024						
10-05-2024						
11-05-2024				1		1
12-05-2024				18		18
13-05-2024				1		1
14-05-2024				1		1
15-05-2024				5		5
16-05-2024				3		3
17-05-2024				7		7
18-05-2024				8		8
19-05-2024				8		8
20-05-2024	1			2		3
21-05-2024	3					3
22-05-2024	14	1				15
23-05-2024	1					1
24-05-2024						
25-05-2024			1			1
26-05-2024		1	3			4
27-05-2024		5	4			9
28-05-2024			1		5	6
29-05-2024					4	4

*Total studied = 120.
